# Supplementary material for: Nationwide Trends in Demographics, Comorbidities, and Mortality Among Elderly Patients with Heart Failure with Preserved Ejection Fraction Hospitalized with Cardiac Arrest
Source: J Pers Med. 2025 Nov 18;15(11):559. doi: 10.3390/jpm15110559 (PMC12653484; doi:10.3390/jpm15110559)
Supplement: Supplementary file 1 [file jpm-15-00559-s001.zip › jpm-3900323-supplementary.pdf]

Supplemental Table S1. Cardiac Arrest and In-Hospital Mortality by Sex Among Patients Hospitalized With HFpEF, 2016–2020. This table presents the number and percentage of cardiac arrest events and in-hospital deaths among hospitalized patients with HFpEF, stratified by sex and year from 2016 to 2020. For each sex group, total hospitalizations are categorized into those with and without cardiac arrest, along with corresponding percentages. Among patients who experienced cardiac arrest, the table further reports the number and percentage who survived or died during hospitalization. The data illustrate temporal trends and sex-based differences in cardiac arrest occurrence and related in-hospital mortality.

Sex	Year	No Cardiac Arrest (n)	% No Cardiac Arrest	Cardiac Arrest (n)	% Cardiac Arrest	Total (n)	No Death (n)	% No Death	Death (n)	% Death	Total Deaths (n)
Male	2016	483,355	98.7%	6,245	1.3%	489,600	2,660	42.6%	3,580	57.4%	6,240
	2017	558,220	98.6%	7,835	1.4%	566,055	3,385	43.3%	4,435	56.7%	7,820
	2018	612,710	98.6%	8,670	1.4%	621,380	3,745	43.2%	4,925	56.8%	8,670
	2019	678,435	98.7%	9,065	1.3%	687,500	3,835	42.3%	5,230	57.7%	9,065
	2020	641,780	98.3%	10,965	1.7%	652,745	4,265	38.9%	6,700	61.1%	10,965
Total		2,974,500	98.6%	42,780	1.4%	3,017,280	17,890	41.8%	24,870	58.2%	42,760
Female	2016	809,664	99.0%	7,785	1.0%	817,449	3,195	41.1%	4,585	58.9%	7,780
	2017	908,215	99.0%	9,495	1.0%	917,710	3,805	40.1%	5,690	59.9%	9,495
	2018	967,595	99.0%	10,245	1.0%	977,840	4,170	40.7%	6,075	59.3%	10,245
	2019	1,045,675	99.0%	10,765	1.0%	1,056,440	4,660	43.3%	6,105	56.7%	10,765
	2020	938,255	98.7%	12,365	1.3%	950,620	4,660	37.7%	7,700	62.3%	12,360
Total		4,669,404	98.9%	50,655	1.1%	4,720,059	20,490	40.5%	30,155	59.5%	50,645

Supplemental Table S2. Cardiac Arrest and In-Hospital Mortality by Race and Year Among Patients Hospitalized With HFpEF, 2016–2020. This table displays the number and percentage of cardiac arrest events and in-hospital deaths among hospitalized patients with HFpEF, stratified by race and year from 2016 to 2020. For each group, total hospitalizations are broken down into those with and without cardiac arrest, along with corresponding percentages. Among patients who experienced cardiac arrest, the table further details the number and percentage who survived or died during hospitalization. Data highlight temporal trends and racial differences in both cardiac arrest incidence and associated in-hospital mortality.

Race	Year	No Cardiac Arrest (n)	% No Cardiac Arrest	Cardiac Arrest (n)	% Cardiac Arrest	Total (n)	No Death (n)	% No Death	Death (n)	% Death	Total Deaths (n)
Black	2016	981,309	99.1%	9,250	0.9%	990,559	3,840	41.6%	5,400	58.4%	9,240
	2017	1,110,579	99.0%	11,705	1.0%	1,122,284	4,915	42.0%	6,780	58.0%	11,695
	2018	1,195,545	98.9%	12,720	1.1%	1,208,265	5,235	41.2%	7,485	58.8%	12,720
	2019	1,313,155	99.0%	13,245	1.0%	1,326,400	5,755	43.5%	7,490	56.5%	13,245
	2020	1,194,695	98.7%	15,155	1.3%	1,209,850	5,935	39.2%	9,220	60.8%	15,155
	Total	5,795,283	98.9%	62,075	1.1%	5,857,358	25,680	41.4%	36,375	58.6%	62,055
White	2016	142,840	98.3%	2,520	1.7%	145,360	1,085	43.1%	1,435	56.9%	2,520
	2017	163,375	98.2%	3,000	1.8%	166,375	1,220	40.7%	1,775	59.3%	2,995
	2018	178,815	98.3%	3,060	1.7%	181,875	1,275	41.7%	1,785	58.3%	3,060
	2019	199,165	98.3%	3,535	1.7%	202,700	1,510	42.7%	2,025	57.3%	3,535
	2020	188,190	97.7%	4,360	2.3%	192,550	1,615	37.1%	2,740	62.9%	4,355
	Total	872,385	98.1%	16,475	1.9%	888,860	6,705	40.7%	9,760	59.3%	16,465
Hispanic	2016	73,115	98.6%	1,070	1.4%	74,185	400	37.4%	670	62.6%	1,070
	2017	88,375	98.7%	1,175	1.3%	89,550	430	36.6%	745	63.4%	1,175
	2018	102,890	98.5%	1,580	1.5%	104,470	745	47.2%	835	52.8%	1,580
	2019	102,230	98.6%	1,450	1.4%	103,680	670	46.2%	780	53.8%	1,450
	2020	94,570	98.0%	1,895	2.0%	96,465	655	34.6%	1,240	65.4%	1,895

	Total	461,180	98.5%	7,170	1.5%	468,350	2,900	40.4%	4,270	59.6%	7,170
Asian or Pacific Islander	2016	25,315	98.6%	360	1.4%	25,675	155	43.1%	205	56.9%	360
	2017	30,625	98.5%	455	1.5%	31,080	150	33.0%	305	67.0%	455
	2018	33,820	98.3%	575	1.7%	34,395	220	38.3%	355	61.7%	575
	2019	37,450	98.4%	590	1.6%	38,040	200	33.9%	390	66.1%	590
	2020	32,960	97.9%	695	2.1%	33,655	250	36.0%	445	64.0%	695
	Total	160,170	98.4%	2,675	1.6%	162,845	975	36.4%	1,700	63.6%	2,675
Native American	2016	4,105	99.0%	40	1.0%	4,145	15	37.5%	25	62.5%	40
	2017	5,140	98.9%	55	1.1%	5,195	15	27.3%	40	72.7%	55
	2018	5,940	98.9%	65	1.1%	6,005	20	30.8%	45	69.2%	65
	2019	6,830	98.8%	80	1.2%	6,910	30	37.5%	50	62.5%	80
	2020	6,540	98.1%	125	1.9%	6,665	45	36.0%	80	64.0%	125
	Total	28,555	98.7%	365	1.3%	28,920	125	34.2%	240	65.8%	365

Supplemental Table S3. Cardiac Arrest and In-Hospital Mortality by Median Household Income Quartile and Year Among Patients Hospitalized With HFpEF, 2016–2020. This table summarizes the incidence of cardiac arrest and in-hospital mortality by year and by national income quartile based on the patient's ZIP Code. For each income quartile (0–25th, 26–50th, 51–75th, and 76–100th), the number and percentage of patients experiencing cardiac arrest are presented, along with outcomes (survived vs. died) among those who experienced cardiac arrest. Cardiac arrest incidence was significantly associated with income quartile ($p < 0.001$). In-hospital mortality also varied by income group, with statistically significant differences for the 0–25th percentile ($p < 0.001$), 26–50th percentile ($p = 0.005$), and 76–100th percentile ($p = 0.009$). Differences in mortality by year within the 51–75th percentile group were not statistically significant ($p = 0.117$). Asterisks in the table indicate groups where $p \geq 0.001$.

Income Quartile (ZIP Code)	Year	No Cardiac Arrest (n)	% No Cardiac Arrest	Cardiac Arrest (n)	% Cardiac Arrest	Total (n)	No Death (n)	% No Death	Death (n)	% Death	Total Deaths (n)
0–25th percentile	2016	351,334	98.8%	4,405	1.2%	355,739	1,880	42.7%	2,525	57.3%	4,405
	2017	391,170	98.7%	5,325	1.3%	396,495	2,245	42.2%	3,080	57.8%	5,325
	2018	409,450	98.7%	5,430	1.3%	414,880	2,190	40.3%	3,240	59.7%	5,430
	2019	464,610	98.7%	5,980	1.3%	470,590	2,510	42.0%	3,470	58.0%	5,980
	2020	426,425	98.3%	7,335	1.7%	433,760	2,680	36.5%	4,655	63.5%	7,335
	Total	2,042,989	98.6%	28,475	1.4%	2,071,464	11,505	40.4%	16,970	59.6%	28,475
26–50th percentile	2016	330,395	99.0%	3,335	1.0%	333,730	1,370	41.1%	1,965	58.9%	3,335
	2017	390,850	98.9%	4,490	1.1%	395,340	1,855	41.5%	2,620	58.5%	4,475
	2018	424,065	98.8%	5,225	1.2%	429,290	2,270	43.4%	2,955	56.6%	5,225
	2019	437,615	98.9%	5,035	1.1%	442,650	2,085	41.4%	2,950	58.6%	5,035
	2020	435,145	98.5%	6,655	1.5%	441,800	2,585	38.8%	4,070	61.2%	6,655
	Total	2,018,070	98.8%	24,740	1.2%	2,042,810	10,165	41.1%	14,560	58.9%	24,725
51–75th percentile	2016	319,555	99.0%	3,340	1.0%	322,895	1,395	41.8%	1,940	58.2%	3,335
	2017	355,740	98.9%	4,040	1.1%	359,780	1,640	40.6%	2,400	59.4%	4,040
	2018	395,285	98.9%	4,370	1.1%	399,655	1,850	42.3%	2,520	57.7%	4,370
	2019	436,605	98.9%	4,700	1.1%	441,305	2,185	46.5%	2,515	53.5%	4,700
	2020	377,005	98.7%	5,015	1.3%	382,020	1,980	39.5%	3,035	60.5%	5,015
	Total	1,884,190	98.9%	21,465	1.1%	1,905,655	9,050	42.2%	12,410	57.8%	21,460
76–100th percentile*	2016	275,170	99.0%	2,780	1.0%	277,950	1,155	41.6%	1,620	58.4%	2,775
	2017	310,045	99.0%	3,220	1.0%	313,265	1,375	42.7%	1,845	57.3%	3,220
	2018	330,935	98.9%	3,640	1.1%	334,575	1,475	40.5%	2,165	59.5%	3,640

2019	363,960	99.0%	3,840	1.0%	367,800	1,585	41.3%	2,255	58.7%	3,840
2020	322,200	98.8%	4,055	1.2%	326,255	1,580	39.0%	2,470	61.0%	4,050
Total	1,602,310	98.9%	17,535	1.1%	1,619,845	7,170	40.9%	10,355	59.1%	17,525

Supplemental Table S4. Cardiac Arrest and In-Hospital Mortality by Primary Payer Type and Year Among Patients Hospitalized HFpEF, 2016–2020. This table presents the number and percentage of cardiac arrest and in-hospital death events, stratified by year and primary payer type (Medicare, Medicaid, Private Insurance, Self-pay/Other). Across all payer groups, the proportion of patients who experienced cardiac arrest and subsequent in-hospital mortality are displayed by year. Cardiac arrest incidence was significantly associated with payer type ($p < 0.001$ for all except Self-pay/Other: $p = 0.110$). In-hospital mortality differences were also significant for Medicare ($p < 0.001$), Medicaid ($p = 0.001$), and Private Insurance ($p = 0.002$), but not for Self-pay/Other ($p = 0.278$). Asterisks in the table indicate payer categories with $p \geq 0.001$ for cardiac arrest or mortality comparisons.

Payer Type	Year	No Cardiac Arrest (n)	% No Cardiac Arrest	Cardiac Arrest (n)	% Cardiac Arrest	Total (n)	No Death (n)	% No Death	Death (n)	% Death	Total Deaths (n)
Medicare	2016	1,187,594	99.0%	12,555	1.0%	1,200,149	5,265	42.0%	7,285	58.0%	12,550
	2017	1,347,424	98.8%	15,725	1.2%	1,363,149	6,455	41.1%	9,265	58.9%	15,720
	2018	1,448,070	98.8%	17,010	1.2%	1,465,080	7,040	41.4%	9,970	58.6%	17,010
	2019	1,571,345	98.9%	17,620	1.1%	1,588,965	7,505	42.6%	10,115	57.4%	17,620
	2020	1,420,880	98.6%	20,635	1.4%	1,441,515	7,940	38.5%	12,690	61.5%	20,630
	Total	6,975,313	98.8%	83,545	1.2%	7,058,858	34,205	40.9%	49,325	59.1%	83,530
Medicaid	2016	16,640	98.5%	250	1.5%	16,890	115	46.0%	135	54.0%	250
	2017	19,465	98.7%	265	1.3%	19,730	115	43.4%	150	56.6%	265
	2018	21,785	98.7%	280	1.3%	22,065	105	37.5%	175	62.5%	280
	2019	22,980	98.6%	335	1.4%	23,315	160	47.8%	175	52.2%	335
	2020	22,540	98.0%	460	2.0%	23,000	145	31.5%	315	68.5%	460
	Total	103,410	98.5%	1,590	1.5%	105,000	640	40.3%	950	59.7%	1,590
Private Insurance	2016	68,635	98.6%	965	1.4%	69,600	365	38.0%	595	62.0%	960
	2017	75,710	98.6%	1,055	1.4%	76,765	480	45.7%	570	54.3%	1,050
	2018	84,430	98.6%	1,220	1.4%	85,650	605	49.6%	615	50.4%	1,220
	2019	97,115	98.6%	1,405	1.4%	98,520	640	45.6%	765	54.4%	1,405
	2020	99,985	98.4%	1,575	1.6%	101,560	600	38.1%	975	61.9%	1,575
	Total	425,875	98.6%	6,220	1.4%	432,095	2,690	43.3%	3,520	56.7%	6,210
Self-pay/Other*	2016	4,955	97.9%	105	2.1%	5,060	40	38.1%	65	61.9%	105
	2017	5,800	99.2%	45	0.8%	5,845	30	66.7%	15	33.3%	45
	2018	6,165	98.1%	120	1.9%	6,285	40	33.3%	80	66.7%	120
	2019	8,555	98.7%	110	1.3%	8,665	60	54.5%	50	45.5%	110
	2020	7,435	98.6%	105	1.4%	7,540	30	28.6%	75	71.4%	105
	Total	32,910	98.5%	485	1.5%	33,395	200	41.2%	285	58.8%	485

Supplemental Table S5. Cardiac Arrest and In-Hospital Mortality by U.S. Hospital Region and Year Among Patients Hospitalized HFpEF, 2016–2020. This table presents the number and percentage of patients with and without cardiac arrest, and the number and percentage of patients who died during

hospitalization, stratified by U.S. census region (Northeast [NE], Midwest [MW], South, West) and year. All cardiac arrest comparisons were statistically significant across regions ($p < 0.001$). For in-hospital mortality, p-values were as follows: Midwest ($p = 0.002$), South ($p < 0.001$), West ($p < 0.001$), and Northeast ($p < 0.001$). Asterisks (*) are not included as all p-values were below the significance threshold of 0.001.

Region	Year	No Cardiac Arrest (n)	% No Cardiac Arrest	Cardiac Arrest (n)	% Cardiac Arrest	Total (n)	No Death (n)	% No Death	Death (n)	% Death	Total Deaths (n)
NE	2016	292,365	99.1%	2,575	0.9%	294,940	1,055	41.1%	1,510	58.9%	2,565
	2017	327,465	99.1%	2,995	0.9%	330,460	1,275	42.6%	1,715	57.4%	2,990
	2018	346,020	99.0%	3,325	1.0%	349,345	1,420	42.7%	1,905	57.3%	3,325
	2019	370,806	99.1%	3,525	0.9%	374,331	1,540	43.7%	1,985	56.3%	3,525
	2020	327,059	98.7%	4,205	1.3%	331,264	1,505	35.8%	2,695	64.2%	4,200
	Total	1,663,715	99.0%	16,625	1.0%	1,680,340	6,795	40.9%	9,810	59.1%	16,605
MW*	2016	334,520	99.1%	3,070	0.9%	337,590	1,220	39.7%	1,850	60.3%	3,070
	2017	382,025	98.9%	4,275	1.1%	386,300	1,805	42.3%	2,465	57.7%	4,270
	2018	410,665	98.9%	4,615	1.1%	415,280	1,935	41.9%	2,680	58.1%	4,615
	2019	449,975	98.9%	4,805	1.1%	454,780	2,160	45.0%	2,645	55.0%	4,805
	2020	414,720	98.7%	5,335	1.3%	420,055	2,140	40.1%	3,195	59.9%	5,335
	Total	1,991,905	98.9%	22,100	1.1%	2,014,005	9,260	41.9%	12,835	58.1%	22,095
South	2016	462,604	98.7%	5,985	1.3%	468,589	2,580	43.1%	3,405	56.9%	5,985
	2017	521,431	98.7%	7,085	1.3%	528,516	2,825	39.9%	4,255	60.1%	7,080
	2018	570,226	98.7%	7,460	1.3%	577,686	3,280	44.0%	4,180	56.0%	7,460
	2019	625,310	98.7%	8,140	1.3%	633,450	3,405	41.8%	4,735	58.2%	8,140
	2020	582,325	98.3%	9,955	1.7%	592,280	3,945	39.6%	6,010	60.4%	9,955
	Total	2,761,896	98.6%	38,625	1.4%	2,800,521	16,035	41.5%	22,585	58.5%	38,620
West	2016	204,145	98.8%	2,405	1.2%	206,550	1,005	41.8%	1,400	58.2%	2,405
	2017	235,545	98.8%	2,975	1.2%	238,520	1,285	43.2%	1,690	56.8%	2,975
	2018	253,415	98.6%	3,515	1.4%	256,930	1,280	36.4%	2,235	63.6%	3,515
	2019	278,045	98.8%	3,360	1.2%	281,405	1,390	41.4%	1,970	58.6%	3,360
	2020	256,005	98.5%	3,835	1.5%	259,840	1,335	34.8%	2,500	65.2%	3,835
	Total	1,227,155	98.7%	16,090	1.3%	1,243,245	6,295	39.1%	9,795	60.9%	16,090

Supplemental Table S6. Cardiac Arrest and In-Hospital Mortality by Rural versus Urban Status and Year Among Patients Hospitalized With HFpEF, 2016–2020. This table summarizes counts and percentages of patients experiencing cardiac arrest and in-hospital death, stratified by rural or urban hospital location and year. All cardiac arrest comparisons were statistically significant with $p < 0.001$. For in-hospital mortality, the overall comparison yielded $p = 0.001$.

Location	Year	No Cardiac Arrest (n)	% No Cardiac Arrest	Cardiac Arrest (n)	% Cardiac Arrest	Total (n)	No Death (n)	% No Death	Death (n)	% Death	Total Deaths (n)
**Rural**	2016	132,845	99.2%	1,035	0.8%	133,880	440	42.5%	595	57.5%	1,035
	2017	149,034	99.2%	1,195	0.8%	150,229	440	36.8%	755	63.2%	1,195
	2018	156,099	99.2%	1,265	0.8%	157,364	480	37.9%	785	62.1%	1,265
	2019	167,950	99.3%	1,250	0.7%	169,200	510	40.8%	740	59.2%	1,250
	2020	154,340	99.0%	1,530	1.0%	155,870	515	33.7%	1,015	66.3%	1,530
	Total	760,268	99.2%	6,275	0.8%	766,543	2,385	38.0%	3,890	62.0%	6,275
**Urban**	2016	1,160,789	98.9%	13,000	1.1%	1,173,789	5,420	41.7%	7,570	58.3%	12,990

2017	1,317,430	98.8%	16,135	1.2%	1,333,565	6,750	41.9%	9,370	58.1%	16,120
2018	1,424,226	98.8%	17,650	1.2%	1,441,876	7,435	42.1%	10,215	57.9%	17,650
2019	1,556,185	98.8%	18,580	1.2%	1,574,765	7,985	43.0%	10,595	57.0%	18,580
2020	1,425,769	98.5%	21,800	1.5%	1,447,569	8,410	38.6%	13,385	61.4%	21,795
Total	6,884,399	98.7%	87,165	1.3%	6,971,564	36,000	41.3%	51,135	58.7%	87,135

Supplemental Table S7. Trends in Comorbidities, Outcomes, and Disposition Among Elderly Patients with HFpEF and Cardiac Arrest, 2016–2020. This table displays the yearly prevalence of key comorbidities, in-hospital outcomes, and post-discharge disposition among elderly patients (≥65 years) hospitalized with HFpEF and cardiac arrest. Continuous variables are reported as median [IQR]. P values for trends were derived using Kruskal-Wallis tests for continuous variables and Pearson Chi-square tests for categorical variables. Significant temporal changes ($p < 0.05$) were observed for most variables, reflecting an increasing burden of chronic disease and shifts in post-acute care needs over time.

Comorbidity	2016	2017	2018	2019	2020	Overall (%)
Diabetes (without complications)	17.8%	10.4%	8.2%	7.9%	7.3%	9.8%
Diabetes (with complications)	28.0%	36.4%	41.2%	39.9%	42.9%	38.5%
Hypertension (complicated)	54.4%	76.9%	79.6%	80.0%	81.8%	75.9%
Hypertension (uncomplicated)	25.8%	4.4%	1.4%	1.0%	1.0%	5.4%
Hyperlipidemia	46.5%	45.0%	49.4%	50.8%	51.0%	48.8%
Chronic pulmonary disease	40.3%	40.0%	41.2%	40.2%	38.0%	39.8%
Chronic kidney disease (CKD)	51.8%	52.7%	51.6%	51.7%	51.6%	51.8%
Obesity	21.8%	22.6%	23.9%	23.1%	25.2%	23.5%
Peripheral vascular disease	15.2%	13.5%	13.6%	13.0%	12.0%	13.3%
Prior myocardial infarction	10.6%	9.7%	10.1%	9.9%	8.3%	9.6%
Prior TIA/stroke	9.4%	8.8%	9.1%	9.2%	8.7%	9.0%
Drug abuse	1.0%	1.0%	0.8%	1.1%	1.1%	1.0%
Alcohol abuse	1.7%	1.5%	2.2%	2.0%	2.3%	2.0%
Depression	9.2%	10.1%	9.6%	9.7%	8.7%	9.4%
Cancer	8.1%	8.1%	8.1%	9.0%	8.1%	8.3%
Prior history of VTE	4.6%	4.5%	4.6%	5.0%	4.4%	4.6%
Hypothyroidism	18.0%	18.1%	19.4%	18.4%	18.0%	18.4%
Other thyroid disorders	1.1%	1.2%	1.0%	1.3%	1.2%	1.2%
Valvular disease	4.3%	4.1%	4.4%	4.1%	4.1%	4.2%

Supplemental Table S8. Trends in Comorbidities, Outcomes, and Disposition Among Elderly Patients with HFpEF and Cardiac Arrest, 2016–2020. This table displays the yearly prevalence of key comorbidities, in-hospital outcomes, and post-discharge disposition among elderly patients (≥65 years) hospitalized with HFpEF and cardiac arrest. Continuous variables are reported as median [IQR]. P values for trends were derived using Kruskal-Wallis tests for continuous variables and Pearson Chi-square tests for categorical variables. Significant temporal changes ($p < 0.05$) were observed for most variables, reflecting an increasing burden of chronic disease and shifts in post-acute care needs over time.

Comorbidity	2016	2017	2018	2019	2020	Overall (%)
Diabetes (without complications)	17.8%	10.4%	8.2%	7.9%	7.3%	9.8%
Diabetes (with complications)	28.0%	36.4%	41.2%	39.9%	42.9%	38.5%
Hypertension (complicated)	54.4%	76.9%	79.6%	80.0%	81.8%	75.9%
Hypertension (uncomplicated)	25.8%	4.4%	1.4%	1.0%	1.0%	5.4%
Hyperlipidemia	46.5%	45.0%	49.4%	50.8%	51.0%	48.8%
Chronic pulmonary disease	40.3%	40.0%	41.2%	40.2%	38.0%	39.8%
Chronic kidney disease (CKD)	51.8%	52.7%	51.6%	51.7%	51.6%	51.8%
Obesity	21.8%	22.6%	23.9%	23.1%	25.2%	23.5%
Peripheral vascular disease	15.2%	13.5%	13.6%	13.0%	12.0%	13.3%
Prior myocardial infarction	10.6%	9.7%	10.1%	9.9%	8.3%	9.6%
Prior TIA/stroke	9.4%	8.8%	9.1%	9.2%	8.7%	9.0%
Drug abuse	1.0%	1.0%	0.8%	1.1%	1.1%	1.0%
Alcohol abuse	1.7%	1.5%	2.2%	2.0%	2.3%	2.0%
Depression	9.2%	10.1%	9.6%	9.7%	8.7%	9.4%
Cancer	8.1%	8.1%	8.1%	9.0%	8.1%	8.3%
Prior history of VTE	4.6%	4.5%	4.6%	5.0%	4.4%	4.6%
Hypothyroidism	18.0%	18.1%	19.4%	18.4%	18.0%	18.4%
Other thyroid disorders	1.1%	1.2%	1.0%	1.3%	1.2%	1.2%
Valvular disease	4.3%	4.1%	4.4%	4.1%	4.1%	4.2%
